# Multiple Chaos Synchronization System for Power Quality Classification in a Power System

**DOI:** 10.1155/2014/902167

**Published:** 2014-02-10

**Authors:** Cong-Hui Huang, Chia-Hung Lin

**Affiliations:** ^1^Department of Automation and Control Engineering, Far-East University, Hsin-Shih, Tainan 744, Taiwan; ^2^Department of Electrical Engineering, Kao-Yuan University, Lu-Chu, Kaohsiung 821, Taiwan

## Abstract

This document proposes multiple chaos synchronization (CS) systems for power quality (PQ) disturbances classification in a power system. Chen-Lee based CS systems use multiple detectors to track the dynamic errors between the normal signal and the disturbance signal, including power harmonics, voltage fluctuation phenomena, and voltage interruptions. Multiple detectors are used to monitor the dynamic errors between the master system and the slave system and are used to construct the feature patterns from time-domain signals. The maximum likelihood method (MLM), as a classifier, performs a comparison of the patterns of the features in the database. The proposed method can adapt itself without the need for adjustment of parameters or iterative computation. For a sample power system, the test results showed accurate discrimination, good robustness, and faster processing time for the detection of PQ disturbances.

## 1. Introduction

The increasing use of electronic equipment can cause electromagnetic disturbances and degrade voltage and current quality. Signal processing, in the time-domain, may have time-varying characteristics [1, 2]. For signal analysis applications, Fast Fourier transformation (FFT), short-time Fourier transformation (STFT), and wavelet transformation (WT) based methods [3–6] have provided promising results in PQ studies. However, the size chosen for the time-window affects both the frequency and time resolution when using a frequency-domain method. Because of the short length of the window at high frequencies and the long length of the window at low frequencies, the frequency-domain method is not suitable for nonstationary signals. The WT, time-frequency domain method, uses cascaded low-pass or high-pass filters and down/upsampling operations to generalize several frequency bands. Specific features render it suitable for the classification of different patterns at specific coefficients in a trial procedure with wavelet decomposition.

Frequency analysis can verify the spectrum characteristics for the classification of PQ disturbances, but the transformation process needs time and requires much memory; so for, online applications, the number of samples is limited. To identify a broad range of features in the time-domain, a dynamic chaos system is a nonlinear deterministic system that constructs mathematical models for systems that are characterized by random signals. This system has some remarkable characteristics, such as heightened sensitivity to initial conditions, broad spectrums of Fourier transforms, and the fractal properties of motion in phase space [7, 8]. It has been extensively studied in many engineering applications, such as adaptive control systems, biomedical signal processing, fluid mechanics, secure communication, and information processing [9–14]. The system consists of a master system (MS) and a slave system (SS). The trajectory of a SS can automatically track the trajectory of a MS. Let MS be a reference, so the dynamic errors are defined as the differences between the SS and the MS and are used to construct various patterns for the classification of PQ disturbances. Therefore, multiple Chen-Lee systems are composed of multiple CS detectors that extract the feature patterns from periodic and nonperiodic signals.

Pattern recognition is important in a detection system, so the artificial intelligence method is designed to classify different types of disturbances, such as multilayer neural networks, wavelet neural networks (WNNs), and support vector machines (SVMs) [4–6, 15, 16]. A classifier uses the maximum likelihood method (MLM) [17–19] to distinguish between different patterns found in the database, with reference to the threshold of rejection. It performs a comparison of the templates of stored patterns and selects the maximum matching likelihood of the pattern above the threshold. It is simpler and less sensitive to the distortion of patterns than traditional methods. It also has a flexible pattern mechanism, with changing training patterns in a dynamic modeling system, to further heighten the accuracy.

For a 14-bus system, the tests demonstrated computational efficiency and accurate recognition. The organization of this paper is as follows: Section 2 addresses the mathematical methods including multiple CS systems and maximum likelihood method and Section 3 describes the implementation of CS system. Sections 4, 5, and 6 present the construction of the multiple CS detectors, experimental results, and conclusions, which show the efficiency of the proposed model.

## 2. Multiple Chaos Synchronization (CS) System

In PQ studies, the disturbances encountered in the process may be time-varying characteristics and voltage fluctuation phenomena, occurring in transient, short-duration, or long-duration variations, involving sag, swell, and harmonic events. The distortion level is investigated and characterized using long-duration recording and statistical events. In many practical applications, FFT and STFT have been applied to analyze transient or short-time fluctuation phenomena. However, when the operating states are partially or totally unavailable, the length chosen for the time-window affects both the frequency and time resolution, so the application of state tracking is restrictive. Therefore, discrete-time, chaotic system is proposed for the tracking of dynamic behaviors, using a computer. With the definition of the master system as a reference, a slave input was designed to synchronously track the dynamic errors, with respect to the reference model. The Chen-Lee system, with known and unknown parameters, shows good symmetrical behavior and has been used in CS applications [20–22]. Generally, it consists of a master system (MS) and a slave system (SS) and can be described as

(1) $$\begin{array}{c} \text{Master System:}\left[\begin{array}{c} {\dot{x}}_{1}\\ {\dot{x}}_{2}\\ {\dot{x}}_{3} \end{array}\right]=\left[\begin{array}{ccc} a & -x_{3} & 0\\ x_{3} & b & 0\\ \frac{1}{3}x_{2} & 0 & c \end{array}\right]\left[\begin{array}{c} x_{1}\\ x_{2}\\ x_{3} \end{array}\right], \end{array}$$

(2) $$\begin{array}{c} \text{Slave System:}\left[\begin{array}{c} {\dot{y}}_{1}\\ {\dot{y}}_{2}\\ {\dot{y}}_{3} \end{array}\right]=\left[\begin{array}{ccc} a & -y_{3} & 0\\ y_{3} & b & 0\\ \frac{1}{3}y_{2} & 0 & c \end{array}\right]\left[\begin{array}{c} y_{1}\\ y_{2}\\ y_{3} \end{array}\right]+\left[\begin{array}{c} u_{1}\\ u_{2}\\ u_{3} \end{array}\right], \end{array}$$

where *X* = [*x*_1_, *x*_2_, *x*_3_] and *Y* = [*y*_1_, *y*_2_, *y*_3_] are state variables, *a*, *b*, and *c* are system parameters, and *U* = [*u*_1_, *u*_2_, *u*_3_] is the nonlinear controller. In control problems, control input *U* is used to cause the dynamic error to decrease to zero, as the time between MS and SS increases. To track dynamic errors, we consider the control term *U* = [*u*_1_, *u*_2_, *u*_3_] = 0. Define the error states *e* = [*e*_1_,*e*_2_,*e*_3_]^*T*^ as

(3) $$\begin{array}{c} e_{1}=x_{1}-y_{2},\;\;e_{2}=x_{2}-y_{2},\;\;e_{3}=x_{3}-y_{3}. \end{array}$$

Then, subtract (2) from (1), and the dynamic-error system can be expressed by

(4) [e˙1e˙2e˙3]=[Φ1Φ2Φ3]=[a−e30e3b013e20c][e1e2e3]=[a−e300b+(e3)2a000c]e.

According to previous studies [20, 23], the system (4) acts as a chaotic attractor that must satisfy the system parameters *a*, *b*, and *c*, for the following specific condition:

(5) $$\begin{array}{c} A>0,\;b<0,\;c<0,\;0<a<-\left(b+c\right). \end{array}$$

The scaling parameters *a*, *b*, and *c* are nonzero constants that control the chaotic motion of the system (4), and phase trajectories indicate that chaotic motion can be controlled. The MS and SS are synchronization such that *e*_1_ = 0, *e*_2_ = 0, and *e*_3_ = 0. For time series analysis, this technique can be used to examine various dynamic errors from the bilateral signals or patterns, such as periodic signals or aperiodic signals in the time-domain.

For computational analysis, the discrete CS systems are used to describe the dynamic rules and time is represented by the integer *n*. Let the error variables be *e*_1_[*i*] = *x*_*m*_[*i*] − *y*_*s*_[*i*], *e*_2_[*i*] = *x*_*m*_[*i* + 1] − *y*_*s*_[*i* + 1], and *e*_3_[*i*] = *x*_*m*_[*i* + 2] − *y*_*s*_[*i* + 2], *i* = 1, 2, 3, …, *n* − 2; the MS and SS are denoted with the subscript *m* and *s*; let *x*_*m*_ be a sequence of sampling data obtained from known signal or training patterns; let *y*_*s*_ be a sequence of sampling data obtained from unknown signal or testing patterns; and *n* is the number of sampling points in one periodic signal. So the general form of dynamic-error system is defined as

(6) $$\begin{array}{c} \left[\begin{array}{c} \Phi_{1i}\\ \Phi_{2i}\\ \Phi_{3i} \end{array}\right]=\left[\begin{array}{ccc} ae_{1}\left[i\right] & -e_{2}\left[i\right]e_{3}\left[i\right] & 0\\ 0 & be_{2}\left[i\right]+\frac{e_{2}\left[i\right]{\left(e_{3}\left[i\right]\right)}^{2}}{a} & 0\\ 0 & 0 & ce_{3}\left[i\right] \end{array}\right]. \end{array}$$

A Chen-Lee based CS detector is used to show that the tracking dynamic errors are zero or have bounded ranges. The dynamic errors Φ_1*i*_, Φ_2*i*_, and Φ_3*i*_ can be calculated using system (6) with system parameters *a*, *b*, and *c*, subject to the constricted condition of (5). System (6) can be used to track the dynamic errors and identify the relevant features of the unknown signals.

**Figure 1:**
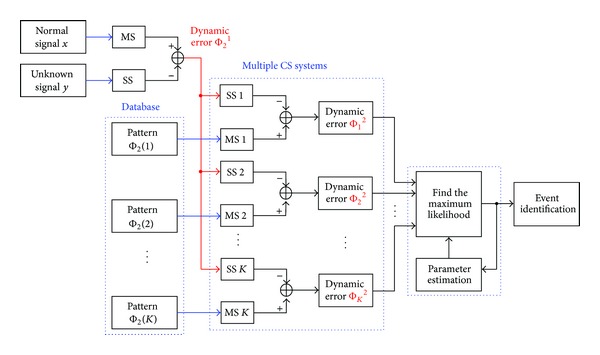
Architecture of multiple chaos synchronization (CS) systems.

The multiple CS systems consist of two stages, as shown in Figure 1. In first stage, it specifies the fundamental signal *x* and unknown signal *y*, cycle by cycle. Then, the dynamic-error equation Φ_2_^1^∈ real number can be used to track the dynamic errors between the starting point, *i* = 1, and the ending point, *i* = *n* − 2, and then testing pattern Φ_2_^1^ connects to each SS. In second stage, we can systematically create training patterns Φ_2_(*k*) as

(7) Φ2(k)=[Φ21(k)Φ22(k)⋮Φ2i(k)⋮Φ2n−2(k)]=b[e2[1]e2[2]⋮e2[i]⋮e2[n−2]] +1a[e2[1]0⋯0⋯00e2[2]⋯0⋯0⋮⋮⋱⋮⋯⋮00⋯e2[i]⋯0⋮⋮⋯⋮⋱⋮00⋯0⋯e2[n−2]] ×[(e3[1])2(e3[2])2⋮(e3[i])2⋮(e3[n−2])2],

where *k* = 1, 2, 3, …, *K* and *K* is the number of PQ disturbances, including harmonics (*har*), voltage sags (*sa*), voltage swells (*sw*), sags or swells involving harmonics (*sah* and *swh*), or momentary interruptions (*int*). With reference to (7), the input of each MS is derived from the *K* training patterns Φ_2_(*k*) = [Φ_21_(*k*), Φ_22_(*k*),…, Φ_2*i*_(*k*),…, *φ*_2*n*−2_(*k*)], as shown in Figure 1.

For each disturbance, we can store *K* training patterns in the database. Input testing pattern Φ_2_^1^, error patterns Φ_*k*_^2^ can be obtained using dynamic-error equation (6). In this study, we choose system parameters *a* = 2, *b* = −3, and *c* = −2 to produce a stable CS attractor. For *m* classes of classification, the likelihood function *L*_*j*_, *j* = 1,2, 3,…, *m*, is defined as [18, 19]

(8) yk=−12σj2(Φk2TΦk2), k=1,2,3,…,Nj,Lj(Φ21Φk2)=∑k=1Njexp⁡(yk)∑j=1m∑k=1Njexp⁡(yk),  K=N1+N2+N3+⋯+Nj.

The covariance *σ*_*j*_^2^ for each class is defined as

(9) σj2=(1/Nj)∑k=1Njwk(Φ(k)TΦ(k))∑k=1NjNjwk,wk=Njm, j=1,2,3,…,m,

where the number of classes is *m*, the number of training patterns in class *j* is *N*_*j*_, and *w*_*k*_ is the weighted value for each class. More training patterns of a class can be continuously stored in the database. For *m* classes classification, the dimension of the vector *y*_*k*_ can be reduced from *K*-dimension to *m*-dimension (*K* > *m*). The likelihood function *L*_*j*_ of the same class will be slightly added in a classifier. Index *L*_*j*_ gives the likelihood that a testing pattern Φ_2_^1^ belongs to training pattern Φ_*k*_^2^ by its value and classification results. The final classification is obtained by choosing the class with the highest probability estimate as

(10) $$\begin{array}{c} L^{*}=\arg\underset{\Phi }{\max}\;L\left({\Phi_{2}}^{1}\right). \end{array}$$

For all of the total likelihoods relating to a hypothesis, the maximum likelihood L* is identified as the correct likelihood.

## 3. Implementation of CS System

**Figure 2:**
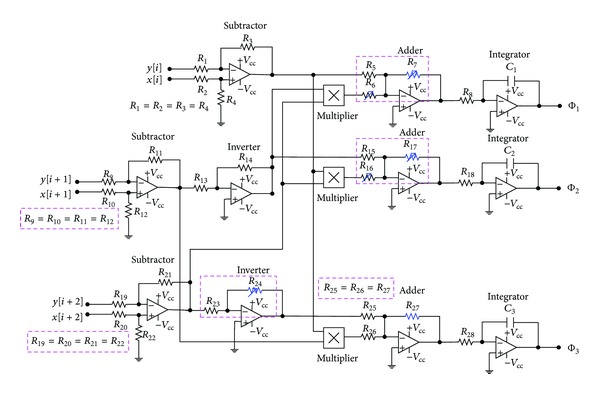
The nonlinear electronic circuits of Chen-Lee based CS system.

A dynamic-error system (4) can be expressed using the integrators and four-fundamental arithmetical operations, including adders, subtractors, and multipliers. We use the dynamic-error equations to implement the CS system, using nonlinear electronic circuits [21, 24], as shown in Figure 2. Using the analog electronic analysis, it can simulate the chaotic Chen-Lee system as an electronic oscillator circuit. The nonlinear circuit uses operational amplifiers (OPAs), resistors, and capacitors. The system parameters (amplifier gains) can be adjusted by

(11) $$\begin{array}{c} \frac{de_{1}}{dt}=\frac{R_{7}}{R_{5}}e_{1}-e_{2}e_{3}, \end{array}$$

(12) $$\begin{array}{c} \frac{de_{2}}{dt}=e_{1}e_{3}-\frac{R_{17}}{R_{15}}e_{2}, \end{array}$$

(13) $$\begin{array}{c} \frac{de_{3}}{dt}=\frac{R_{27}}{R_{26}}e_{1}e_{2}+\frac{R_{24}}{R_{23}}e_{3}, \end{array}$$

(14) $$\begin{array}{c} a=\frac{R_{7}}{R_{5}}=2,\;\;\left|b\right|=\frac{R_{17}}{R_{15}}=3,\;\;\left|c\right|=\frac{R_{24}}{R_{23}}=2, \end{array}$$

(15) $$\begin{array}{c} R_{6}=R_{7},\;R_{16}=R_{17},\;\frac{R_{27}}{R_{26}}=\frac{1}{3}. \end{array}$$

We can estimate the values of resistors *R*_5_, *R*_7_, *R*_15_, *R*_17_, *R*_23_, and *R*_24_ by using (14) and satisfying specific condition (5). A nonlinear electronic circuit is a chaotic attractor, so the default initial conditions are zero. The phase diagrams can be plotted for the time interval 1/60 seconds. The time scale is

(16) $$\begin{array}{c} t=R_{8}C_{1}n=R_{18}C_{2}n=R_{28}C_{3}n=RCn, \end{array}$$

where *n* is the number of sampling points.

**Figure 3:**
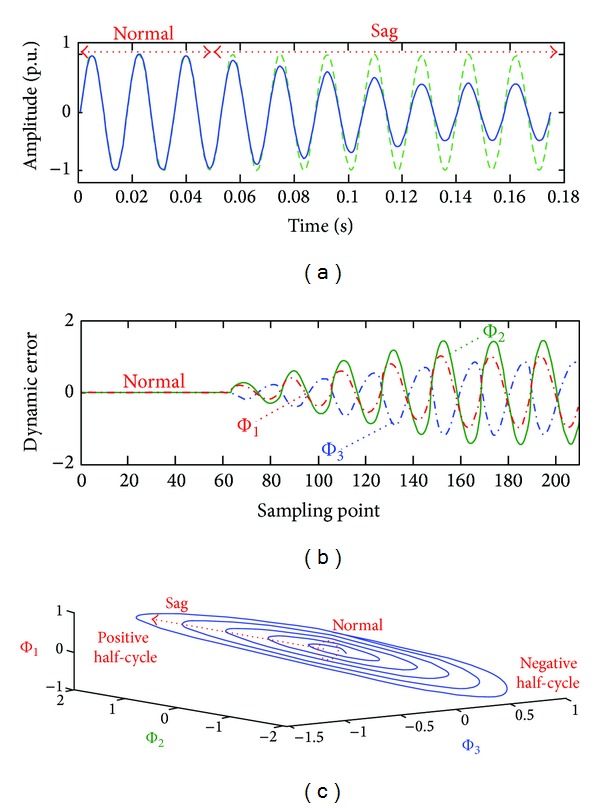
Experimental simulation results. (a) Coupling signal: ideal periodic voltage signal and voltage sag signal, (b) dynamic errors Φ_1_, Φ_2_, and Φ_3_, and (c) phase diagram of CS system.

For the system parameters *a* = 2, *b* = −3, and *c* = −2 and the initial conditions as *x*_1_ = *x*_2_ = *x*_3_ = 0, and *y*_1_ = *y*_2_ = *y*_3_ = 0, the phase diagrams of the simulation experimental results are shown in Figure 3. One of the two periodic signals is the ideal voltage signal with 60 Hz-fundamental frequency and the other is the voltage sag signal, as shown in Figure 3(a). Voltage sags are 70% to 90% reductions in the rated voltage with short-duration variations. The sampling rate is *f*_*s*_ = 1.44 kHz, and the number of sample points is 24 (*n* = 24, 12 points in the positive half-cycle, and 12 points in the negative half-cycle). The Chen-Lee based CS detector acts to track the dynamic errors between the fundamental and sag signals. The dynamic errors Φ_1_, Φ_2_, and Φ_3_ reflect the chaotic behaviors and control the motions in a bounded region with the specific parameters *a*, *b*, and *c*. As can be seen, from the phase diagram in Figure 3(b), the dynamic errors increase as voltage sag increases, and the trajectories of the chaotic motions increases. Each trajectory is bounded within an elliptical region, Φ_1_: ±1.0, Φ_2_: ±2.0 and Φ_3_: ±1.0, as shown in Figure 3(c). The dynamic errors increase as the distortions gradually become pronounced, especially the dynamic error Φ_2_. The same phenomena occur in other PQ disturbances. Therefore, the dynamic error Φ_2_ was used to construct various patterns for the classification of PQ disturbances.

## 4. Multiple CS Detectors Construction

PQ disturbances occur in many levels of distortion and waveforms. Voltage fluctuation phenomena, such as voltage swell and voltage sag, transient overvoltage, and voltage interruption, can occur due to a lightning strike, capacitor switching, a large motor starting, nearby circuit faults, or artificial accidents and can lead to power interruptions. These disturbances are caused by equipment interaction and are exacerbated by wiring and grounding problems. These voltage fluctuations are low voltage, high voltage, or voltage interruption at the fundamental frequency voltage within short durations (0.5 seconds~1 minute) or long-duration (>1 minute). Harmonics are long duration voltage fluctuations in low or high frequency components and are caused mostly by electronic equipment. The common types of disturbances and their impacts are listed below [1].

- Voltage sag (dip) is a sudden voltage drop of 10~90% in magnitude. It often lasts for one-half cycle to 1 minute. When the voltage drops by 30% or more, we consider the system status to be severe, such as a fault in a power system or switching of heavy loads.
- Voltage swell is a voltage rise above 110% of the normal voltage for the duration of between one-half cycle and 1 minute. It has been known to cause a single line-to-ground fault on the system, a failure of marginal components in electronic equipment, and large loads or capacitors are removed.
- Interruptions can be classified as momentary or sustained voltage interruptions. An interruption occurs when the supply voltage drops to less than 90% of the normal voltage for the duration of one-half cycle to 5 minutes. These events can be the result of faults, equipment failures, or control malfunctions.
- Harmonics are fundamental voltages that have integer multiples of the frequency. Total harmonic distortion (THD) levels exceed the standard [1, 2], due to the nonlinear characteristics of power electronic equipment and loads on the power system.

**Figure 4:**
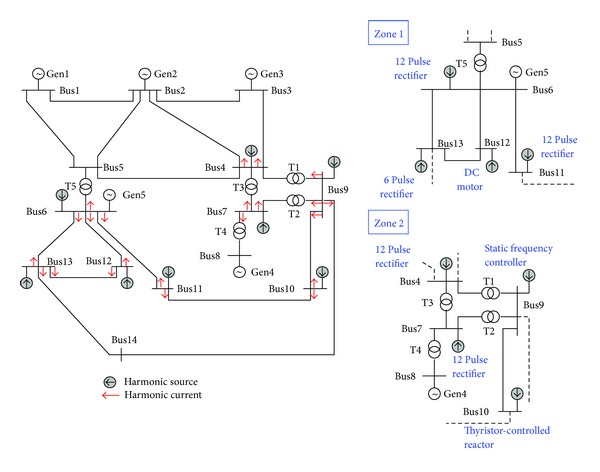
One-line diagram of the 14-bus power system.

A 14-bus system was used for the test example, as shown in Figure 4. The system has 5 generator buses, 15 lines, and 5 transformers. There are 8 harmonic sources in Zone 1 (Buses 6, 11, 12, and 13) and Zone 2 (Buses 4, 7, 9, and 10), including power rectifiers or converters.  Table 1 shows the harmonic current components of each harmonic source, obtained by the field testing. For each bus, harmonics and voltage fluctuation phenomena were identified, as well as the harmonic source causing voltage distortion in neighboring buses. Using the fundamental power flow, bus voltages could be calculated for each bus to define normal operation with various load combinations and work durations.

Therefore, training data could be collected for normal voltages and voltage fluctuations, such as normal (*nor*), *sa*, *sw*, and *int*. In addition, it was possible to simulate harmonic voltages using harmonic power flow with various harmonic load combinations in Zone 1 or Zone 2, that is, load combinations {Bus13}, {Bus13, Bus6}, {Bus13, Bus11}, {Bus13, Bus12}, {Bus13, Bus6, Bus11}, {Bus13, Bus6, Bus12}, and {Bus13, Bus11, Bus12} at Bus13. Harmonic voltages, such as *har*, *sah*, and *swh*, could also be systematically determined at each bus.

**Figure 5:**
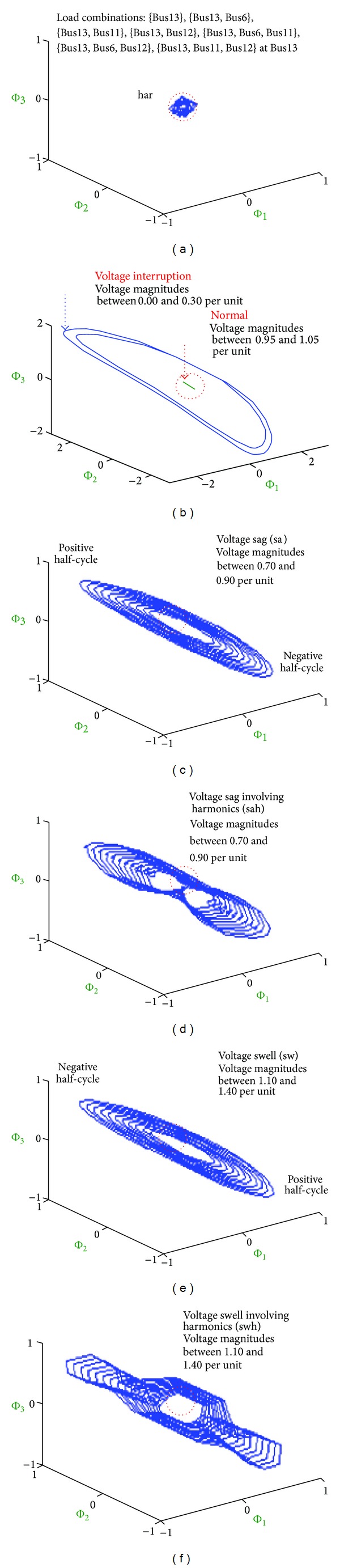
Various butterfly patterns at Bus13. (a) Butterfly patterns for harmonics, (b) butterfly patterns for normal voltage and voltage interruption, (c) and (d) butterfly patterns for sag events, and (e) and (f) butterfly patterns for swell events.

A sampling rate, *f*_*s*_ = 1.44 kHz, was used and the number of sampling points was 24 (*n* = 24, 12 points in the positive half-cycle, and 12 points in the negative half-cycle). According to (6) with *a* = 2, *b* = −3, and *c* = −2, the CS system is a chaotic attractor. Dynamic errors Φ_1*i*_, Φ_2*i*_, and Φ_3*i*_ can be estimated using normal voltages and distorted voltages. Their motion trajectories are in a bounded region within ±2, and show good symmetrical behavior, as shown in Figure 5. These elliptical trajectories follow the so-called butterfly patterns. The motion trajectories of normal voltages describe small ellipses and increase as the variation in voltage magnitudes gradually becomes more pronounced. In addition, the patterns reveal saw tooth and polygonal characteristics for voltage sag or voltage swell involving harmonics.

The scatter diagram of magnitude-duration shows that for Taiwan's science parks and high-tech customers, most magnitudes were distributed between 0.70~0.90 per unit (p.u.) and 1.10~1.30 p.u. within 10~30 cycles [2]. Therefore, this study considers the short- and long-duration voltage fluctuation phenomena. The 61 sets of training patterns for multiple CS detectors at each observation bus, include the following events.

- harmonics (*har*): harmonic load combinations with 7 sets of training patterns, *N*_*har*_ = 7,
- voltage sag (*sa*) or sag involving harmonics (*sah*): voltage reduction between 10 and 30% in magnitude with 22 sets of training patterns, *N*_*sa*_ = 11 and *N*_*sah*_ = 11,
- voltage swell (*sw*) or swell involving harmonics (*swh*): voltage rise between 10 and 30% in magnitude with 22 sets of training patterns, *N*_*sw*_ = 11 and *N*_*swh*_ = 11,
- normal (*nor*): voltage magnitudes between 0.95 and 1.05 p.u. with 7 sets of training patterns, *N*_*nor*_ = 7,
- voltage interruption (*int*): voltage magnitude less than 10% of nominal with 3 sets of training patterns, *N*_*int*_ = 3.

A database was constructed for the 61 training patterns (*K* = 61 in this study) to validate the proposed method. These patterns can be classified into 7 categories. We systematically created template patterns Φ(*k*), *k* = 1, 2, 3,…, 61, such that we had 61 sets of comparative patterns for the MLM based classifier. The final probability *L*_*j*_(Φ/Φ(*k*)) is between 0 and 1, found using (8). Equation (10) was used to find the highest probability. For 7 categories classification, a threshold of rejection was used to confirm the decision. This can be defined as [25]

(17) $$\begin{array}{c} L^{*}>\theta_{\text{judge}}=0.5\times \frac{1}{7}\sum_{j=1}^{7}L_{j}\left(\frac{\Phi }{\Phi \left(k\right)}\right). \end{array}$$

The index L* = arg max⁡ {*L*_*har*_, *L*_*sa*_, *L*_*sah*_, *L*_*sw*_, *L*_*swh*_, *L*_*nor*_,  *L*_*int*_} is maximum one and is up to the threshold value *θ*_judge_. It is as high as possible and approaches to 1. The threshold *θ*_judge_ as (17) provides a degree of confidence for the classification of PQ disturbances.

## 5. Experimental Results

The proposed CS based detector was designed using a PC Pentium-IV (2.4 GHz with 480 MB RAM) and MATLAB software. Studies of harmonic and voltage fluctuation demonstrate the effectiveness of the proposed method.

### 5.1. Voltage Sag and Swell Involving Harmonics

**Figure 6:**
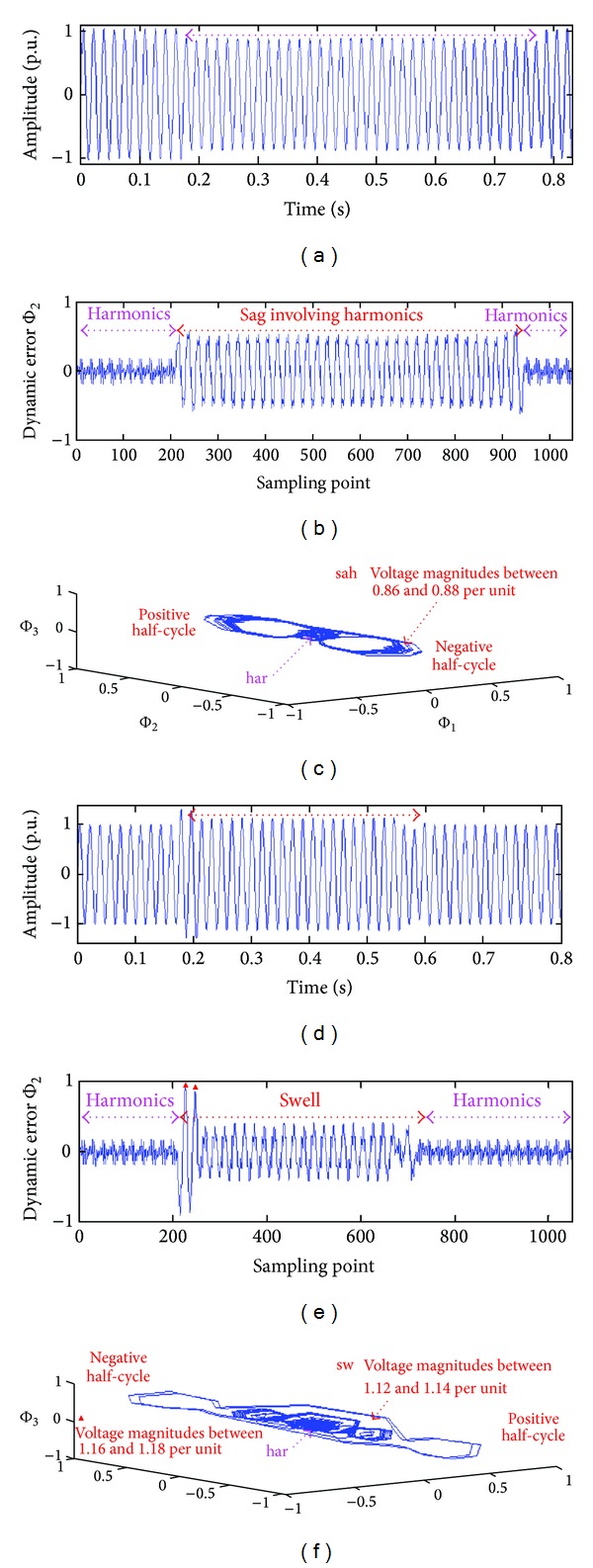
(a) Voltage sag involving harmonics in the time-domain, (b) dynamic errors for *har* and *sah* events, (c) motion trajectories of butterfly patterns for *har* and *sah* events, (d) voltage swell involving harmonics in the time-domain, (e) dynamic errors for *har* and *swh* events, and (f) motion trajectories of butterfly patterns for *har* and *swh* events.

Suppose that there are multiple harmonic sources at Bus6, Bus9, Bus12, and Bus13, and the total harmonic voltage distortion of each bus is greater than 2.5%. If the voltage sags caused by heavy motor loads are at Bus12, then multiple harmonic sources are at the Bus6, Bus9, and Bus13.  Figure 6(a) shows the harmonic voltages and voltage sag within about 30 cycles in the time-domain. When the voltage sag suddenly reaches 86~88% of the rated voltage, the multiple CS detectors acts to track the dynamic errors between the known pattern Φ_2_(*k*) and unknown pattern Φ_2_. As shown in Figure 6(b), the critical dynamic errors at the beginning and ending of events were noted to monitor the voltage sags involving harmonics. As can be seen in Figure 6(c), dynamic errors Φ_1_, Φ_2_, and Φ_3_ are estimated using normal voltage and distorted voltages.

**Figure 7:**
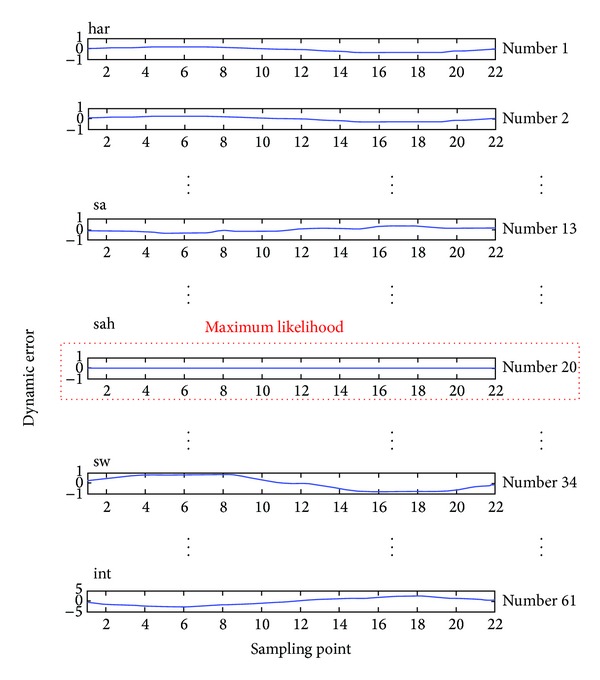
The maximum matching likelihood among different patterns. Note: *har*: Number 1~Number 7, *sa*: Number 8~Number 18, *sah*: Number 19~Number 29, *sw*: Number 30~Number 40, *swh*: Number 41~Number 51, *nor*: Number 52~Number 58, and *int*: Number 59~Number 61.

The butterfly-pattern motion trajectories are bounded inside the elliptical region, Φ_1_: ±0.1, Φ_2_: ±0.2, and Φ_3_: ±0.1 for a *har* event, and Φ_1_: ±0.5, Φ_2_: ±1.0, and Φ_3_: ±0.5 for a *sah* event. When voltage magnitudes suddenly decrease, the phase portraits of Φ_1_-Φ_2_-Φ_3_ exhibit chaotic motion. The various morphologies can also be used to observe PQ disturbances. For 50 detection cycles, the MLM based classifier performed a comparison of the patterns in the database. If pattern Φ_2_ was similar to any patterns Φ_2_(*k*), *k* = 1, 2, 3,…, 61, the dynamic errors approached zero.  Figure 7 shows the maximum matching likelihood between different patterns. The average output was computed using (8), as *L*_ave_ = [0.4722,0.1004,0.1105,0.0980,0.0000,0.0821,0.1368] for a* har* event, and *L*_ave_ = [0.0435, 0.1853, 0.5137, 0.0289, 0.0000, 0.0762, 0.1523] for a *sah* event. For the maximum one L* = argmax {*L*_*har*_, *L*_*sa*_, *L*_*sah*_, *L*_*sw*_, *L*_*swh*_, *L*_*nor*_, *L*_*int*_}, the final outputs indicate the “*har*” and “*sah*,” respectively. This confirms that the proposed method has a higher accuracy for PQ disturbances.

When large loads are suddenly removed at Bus 12, voltage swells rise to 116~118% and 112~114% of the rated voltage. The scenario shown in Figure 6(d) describes voltage swell with magnitudes of up to 110% of the nominal voltage for about 25 cycles in the time-domain. The dynamic errors and butterfly-pattern motion trajectories were also noted to monitor the voltage swells involving harmonics, as shown in Figures 6(e) and 6(f). The average outputs were *L*_ave_ = [0.4723, 0.1008, 0.1134, 0.0984, 0.0000, 0.0745, 0.1406] fora *har* event, and *L*_ave_ = [0.0464, 0.0306, 0.0313, 0.1032, 0.5473, 0.0794, 0.1617] fora *swh* event. The final outputs indicate the “*har*” and “*swh*”, respectively. The simulation results show that the proposed method demonstrates high confidence, for all tests of the classification of PQ disturbances.

### 5.2. Performance Test and Discussion

**Figure 8:**
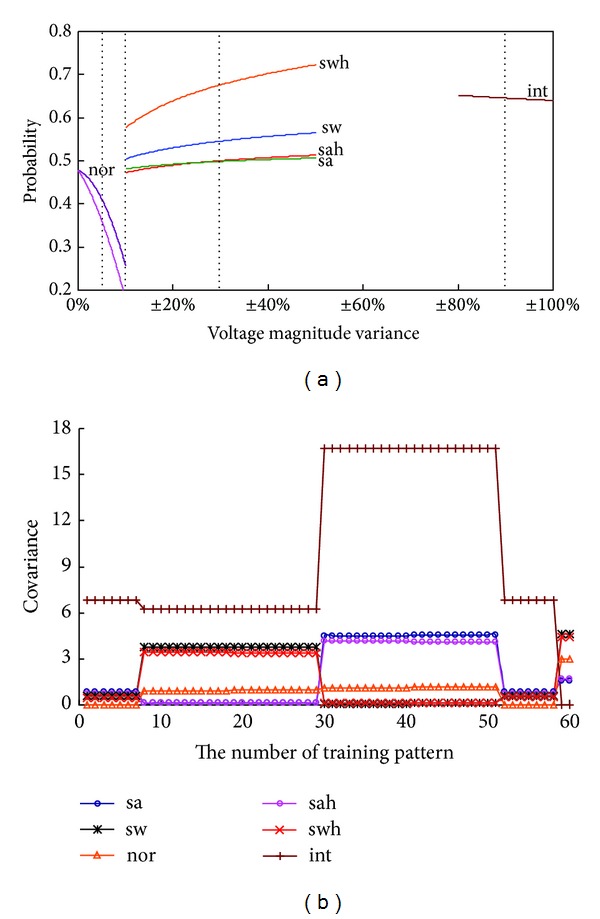
(a) Average probability versus voltage magnitude variant. (b) Covariance versus the number of training pattern for voltage fluctuation phenomena. Note: (1) symbol “+” means voltage magnitude increase and symbol “−” means voltage magnitude decay, (2)  *har*: Number 1~Number 7, *sa*: Number 8~Number 18, *sah*: Number 19~Number 29, *sw*: Number 30~Number 40, *swh*: Number 41~Number 51, *nor*: Number 52~Number 58, and *int*: Number 59~Number 61.

In this study, computer simulation was used to change traced disturbances. Voltage data were produced for varying voltage magnitudes and a variation in harmonic components varying from −50% to +50%, *V*_THD_% ≥ 2.5%.  Figure 8(a) shows the average probability versus voltage magnitude variance for 350 untrained data including *nor*, *sa*, *sah*, *sw*, *swh*, and *int* events. With training patterns for *sa* and *sah* events having a specific sag range between 0.7 and 0.9 per unit, voltage magnitudes between 0.50 and 0.90 per unit were identified as “*sa*” and “*sah*” events. The results can be also observed for “*sw*” and “*swh*” events between 1.10 and 1.50 per unit. Voltage interruptions were gradually identified for a voltage magnitude below 0.20 per unit. With the 61 sets of training patterns, the proposed method could work in a dynamic environment, with voltage fluctuation with harmonics. The experimental results show that the proposed method has high confidence in the classification of all events, using the rejection threshold *θ*_judge_.

To compare the proposed method with the traditional method, a multilayer neural network was also designed as a classifier, using a least mean square (LMS) algorithm. However, the choices of the network architectures, initial network parameters, learning rates, and convergent condition affect learning performances [6]. As the number of training patterns increases (high-dimensional pattern space), the training process and classification efficiency become a bottleneck using the gradient methods, steepest descent methods, or optimization methods. The proposed method was used to determine the maximum matching likelihood. Although the performance of the MLM based method is affected by covariance, values are automatically adjusted by the weighted value and training patterns for various voltage fluctuation phenomena, as shown in Figure 8(b). The proposed method can adapt itself in real-time system applications without the need for parameter assignment or iterative computation.

## 6. Conclusion

The multiple CS detectors for power quality disturbances classification have been examined in this paper. The Chen-Lee based chaos system was used to track the dynamic errors between the normal signal and the distorted signals in the time-domain. The butterfly patterns reveal various morphological motions for harmonics, voltage fluctuation phenomena, and multiple disturbances. These motions can also point out the beginning and ending of disturbance occurrences. Subsequent, multiple CS detectors act to track dynamic errors between feature pattern and training patterns. The maximum likelihood method (MLM) then used these feature patterns to classify PQ disturbances. It can automatically adjust the parameters and could be suitable for application in a dynamical environment. To ensure the continuity service and higher reliability, the proposed method also provides a promising result for fault detection in intelligent microgrid, including short-circuit and line-to-ground faults. Intelligent electronic devices, such as digital protective relays and digital fault recorders, can be implemented through analog electronic circuits, microprocessor, and communication links for further use in protection system.

## Figures and Tables

**Table 1:** Harmonic current components in percentage by field tests.

Bus Bar	3rd	5th	7th	9th	11th	13th	15th	17th	19th	21th	23rd	25th	Nonlinear device
7, 11, 13	1.50	22.0	15.0	0.00	10.2	8.40	0.00	4.30	3.40	0.00	0.59	0.00	6-pulse rectifier
4, 6	0.15	0.55	0.29	0.00	6.20	4.50	0.00	0.10	0.21	0.00	0.46	0.00	12-pulse rectifier
9	0.00	17.0	10.1	0.00	6.10	4.42	0.00	3.83	3.20	0.00	2.58	2.32	Static frequency converter
10	13.8	5.05	2.59	1.57	1.05	0.75	0.57	0.44	0.35	0.29	0.24	0.20	Thyristor-controlled reactor
12	1.20	33.6	1.60	0.00	8.70	1.20	0.00	4.50	1.30	0.00	2.80	0.00	DC Motor
